# An Ethnobotanical Survey of Indigenous Knowledge on Medicinal Plants Used by Communities to Treat Various Diseases around Ensaro District, North Shewa Zone of Amhara Regional State, Ethiopia

**DOI:** 10.1155/2023/5575405

**Published:** 2023-11-01

**Authors:** Mikias Teshome, Firew Kebede, Tamene Yohannes

**Affiliations:** ^1^Ethiopian Biodiversity Institute, Addis Ababa, Ethiopia; ^2^Departments of Biology, Hawassa University, Awasa, Ethiopia

## Abstract

The study was conducted to investigate and document medicinal plants and associated knowledge on the utilization, management, preparation, and way of administration of the medicinal plant resources in Ensaro district, north Shewa zone, Ethiopia. A total of 100 informants were sampled from four study sites, and questionnaire surveys, semistructured interviews, ranking, and transect walk techniques were employed for data collection in midland, lowland, and highland agroecology and natural forests, riverine forests, and farmlands. Vast sources of the traditional healing knowledge of plant species conveyed from one generation to the next by word of mouth were from a family. A total of 101 medicinal plant species were identified from the study site, which belong to 95 genera and 49 families. These medicinal plants are used to treat about 35 types of human ailments. Families Fabaceae and Poaceae were represented by the highest number of medicinal plant species, followed by the *Asteraceae*, *Lamiaceae*, and *Euphorbiaceae* species. Out of the total medicinal plants' species, 46.53% were herbs and 33.66% were shrubs. Most of them have medicinal properties in their leaf, root, seed, bark, stem, latex, sap seed, and fruits. Medicine from these plant parts is prepared in fresh, dried, and both fresh and dried states. The highest informant consensus was documented for the plant *Ocimum lamiifolium* used by 75% of informants for its medicinal value in treating fibril illness. *Cucumis ficifolius* and *Eucalyptus globules* are used by 41% and 39% of informants ranking second and third, respectively, for their medicinal value. This study revealed that indigenous knowledge of traditional medicine is still popular among local communities in the study area. The conservation strategy practiced by local people is not enough to tackle the erosion of plant species from their habitats. Thus, the conservation of these plants and the associated knowledge base is very essential.

## 1. Introduction

Plants are the most essential to human well-being in providing basic human needs. Human beings started using plants for disease control and prevention since time immemorial. According to Martin [1], ethnobotany is a wide term referring to the study of people's classification, management, and use of plants. Early humans acquired knowledge on the utilization of plants for disease prevention and curative purposes through many years of experience, careful observations, and trial and error experiments [1, 2] Such ethnomedicinal knowledge involves traditional diagnosis, collection of raw materials, preparation of remedies, and prescription to the patients. From the evidence provided by Kibebew [3], it is estimated that about 75–90% of the rural population in the world excluding western countries depends on traditional medicines as their only healthcare system.

In general, ethnobotany is the scientific investigation of plants as used in indigenous culture for food, medicine, magic, rituals, building, household utensils and implements, musical instruments, firewood, pesticides, clothing, shelter, and other purposes [4]. There are several plants that possess not only medicinal value but also provide materials for survival, economic, and forage values and preserve cultural heritages, biological information, and indigenous knowledge. For example, forest resources are home to an estimated 60 million indigenous people, who are directly dependent on forest resources and the health of forest ecosystems for their livelihoods [5]. Nature is always a golden sign to show the prominent phenomena of coexistence. Natural products from plants, animals, and minerals are the basis for treating human diseases [6]. Medicinal plants are presently in demand, and their acceptance is increasing progressively [7]. Awareness and application of plants to prepare food and medicine have been realized through trial and error, and gradually, humans were able to meet their needs from their surroundings [8]. Information about medicinal plants has long been transmitted gradually and from generation to generation; this increased knowledge has allowed us to uncover the medicinal properties of plants and their potential benefits for human health. Through scientific research and technological advancements, we have gained a more comprehensive understanding of various fields, including medicine.

Currently, increasingly, scientists and pharmaceutical companies are also looking at traditional knowledge to identify new drugs by combining with modern scientific methods and researchers. So the integration of traditional knowledge and scientific research holds great potential for identifying new drug sources and advancing medical science; herbal drugs can help the emergence of a new era of the healthcare system to treat human diseases in the future. However, in Africa, the traditional knowledge of the utilization of plants was undocumented. Most of the knowledge acquired by local people has been passed on to them by word of mouth from one generation to the other [9]. The majority of people in Ethiopia still depend on traditional medicine mainly due to the shortage of pharmaceuticals, inadequate coverage of the modern medical system, and unaffordable prices of modern drugs [10]. In Ethiopia, little emphasis has been given to ethnobotanical (ethnomedicinal) studies over the past decades [11, 12] even if there has been some attempt to investigate medicinal plants and indigenous knowledge on sustainable use and management of plant resources. The lack of conservation actions and activities is observed in Ensaro woreda (district), which is similar to other areas in Ethiopia. Even though based on the information gathered from Ensaro woreda agricultural extension, it is known that the woreda has relatively better plant resources, and hence, the associated traditional knowledge resource is expected to be significant.

The current plant use trend shows that the environment is facing problems by both natural and anthropogenic factors of resource depletion and loss of indigenous knowledge such as in other areas of the country. Thus, concerted ethnobotanical research plays a vital role in gathering information on plants and related indigenous knowledge for conservation and sustainable utilization. However, to have the full picture of ethnomedicinal knowledge of societies in Ethiopia, geographical, cultural, and botanical diversity studies need to be included. Recently, some studies were conducted on ethnobotanical and associated indigenous knowledge at some localities of Ethiopia but, since Ethiopia has rich plant diversity and diverse ethnolinguistic groups, there is a wide gap in our knowledge about ethnobotanical data and information from various parts of Ethiopia, so it needs a lot of research. Nevertheless, no study was conducted to include medicinal plants and indigenous knowledge of the local communities of Ensaro woreda in the medicinal records of Ethiopia. The present study was conducted to identify the medicinal plants used to treat human ailments in the local communities of Ensaro district, north Shewa zone of Amhara region, Ethiopia.

## 2. Materials and Methods

### 2.1. Description of the Study Area

**Figure 1:**
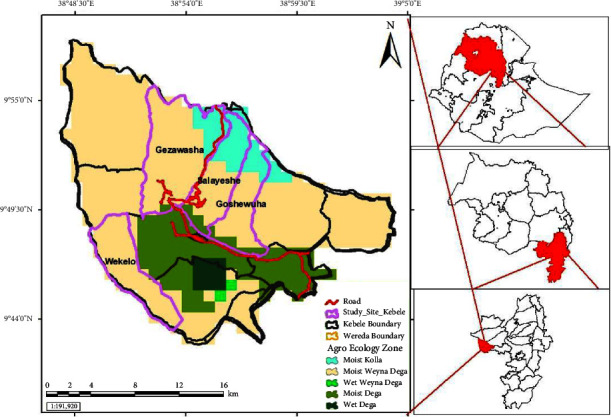
Location study area.

Ensaro woreda is found in the north Shewa zone of Amhara Regional State in Ethiopia. Geographically, the woreda is located between 9° 35′–9° 55′N and 38° 50′–39° 5′E with an average elevation of 2,435 meters above sea level (Figure 1). The woreda has one urban kebele and 13 rural kebeles. The capital city of the woreda is Lemi town which is located 130 km northwest of Addis Ababa and 85 km from Debre Birhan. Based on the 2007 National Census conducted by the Central Statistical Agency (CSA) of Ethiopia, the woreda has a total population of 58,203, of whom 29,888 were male and 28,315 female; 3,164 (5.44%) were urban inhabitants [13]. Based on the study by Abirham [14], Ensaro is bordered in the south and west by the Oromia Region, in the north by Jemma River which separates it from Merhabiete woreda, in the northeast by Moretna Jiru, and in the east by Siyadebrina Wayu woreda. The woreda's total land area is 44,217.6 hectares. Out of these undulating areas, it covers about 50% flat plains, 40% mountainous, and others 10%. According to the woreda administration and community classification, the woreda's agroecology is subdivided into kola (33%), woinadega (46%), and dega (21%).

Ensaro woreda was selected purposively due to the presence of good natural forest and low accessibility of roads and infrastructure. A reconnaissance survey of the study area was conducted from December 29, 2019, to January 4, 2020. Four kebeles, namely, Wokolo with a high altitude located southeast of Lemi town, Gezawasha from a low altitude located west of Lemi town, and Salayesh and Gosh wuha from a medium altitude area located northeast of Lemi, were purposefully selected from the total of 13 kebeles depending on accessibility, vegetation cover, altitude, agroecology, and availability of traditional practitioners.

### 2.2. Climate

**Figure 2:**
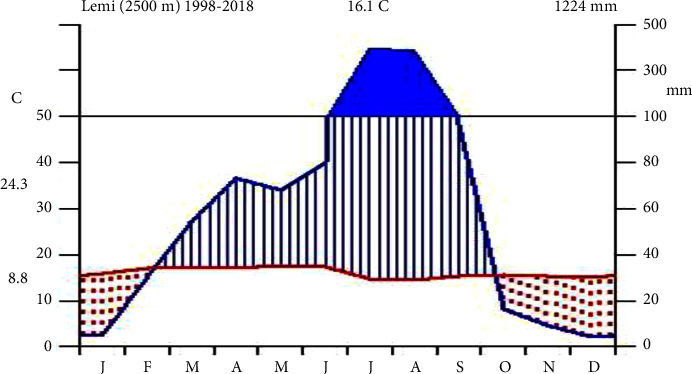
Climadiagram of the study site.

Climate is one of the elements of the physical environment which has a pronounced impact on settlement patterns, human way of life, the type of soil, flora, and fauna that existed and/or developed so forth. Among different climatic elements, temperature and rainfall have a considerable impact in such an agrarian country such as Ethiopia and more actually in the area under study. Twenty years (1998–2018) of meteorological data were taken from the Addis Ababa National Meteorology Service Agency (recorded from Lemi station), indicating that the distribution of the rainfall unimodal (one rainfall peak) pattern obtains high rainfall between June and August and low rainfall in March to May, and the dry season extends from September to February; the evidence has been obtained from the Addis Ababa National Meteorology Service Agency [15]. The mean annual rainfall of the study area within twenty years was 1,224 mm, whereas the lowest mean annual temperature over twenty years was 8.8°C, and the highest was 20°C (Figure 2).

#### 2.2.1. Selection of Informants

A total of 100 informants (80 male and 20 female) aged between 22 and 82 were selected randomly from the selected kebeles. From the total of 100 informants, 20 informants (five key informants from each kebele) were purposefully selected as key informants by using information and recommendations from local healers, elders, kebele administrators, and kebele developmental agents (DAs) by using purposive sampling approaches, respectively, following [1, 16]. The local healer gives priority as key informants.

#### 2.2.2. Ethnobotanical Data Collected

Ethnobotanical data were collected between December and March 2020. Ethnobotanical data were collected in very close interaction with informants using semistructured questions prepared beforehand in English and translated to Amharic (the language of the inhabitants during interview administration). The interviews were based on and around this checklist, and some issues were raised promptly depending on the responses of an informant. All of the interviews were held in Amharic. In a more structured interview, the healers were asked about plants, uses, and the method of preparation of plants, route of administration as well as storage, side effects, contradiction, and antidotes of herbal preparations. Important ethnobotanical information was gathered that was provided by informants.

#### 2.2.3. Specimen Collection

Specimens collected during the guided field walk were pressed, numbered, dried, and given vernacular names on each sheet and dried for identification. Identification of specimens was carried out both in the field and in the herbarium. Identification was also carried out using the Flora of Ethiopia and Eritrea compared with already identified voucher specimens. Finally, the identified specimens were stored at the Ethiopian Biodiversity Institute (EBI) herbarium.

#### 2.2.4. Analysis of Ethnobotanical Data

Data were analysed following the survey and analytical tools for ethnobotanical methods as recommended by Martine, Cotton, and Cunningham [1, 17, 18]. The ethnobotanical data were analysed using quantitative and qualitative methods of data analysis. Descriptive statistics such as percentage, frequency distribution, and graphs were used to analyse the data collected through semistructured, open-ended, and some close-ended questions.

*Preference ranking* was conducted to evaluate the degree of preferences or levels of importance of certain selected plants or parts of plants and following Martine and Cotton [1, 17] by using six of the key informants who were randomly identified and who were invited to rank nine medicinal plant species that are used for the treatment of stomachaches because it is a frequently appearing disease in the study area and informed by several informants, and the ranking was based on the informants' perceptions (Table 1). Accordingly, each informant was assigned the value based on their preference for the plant species that are used. Finally, the total score was identified, and the rank of each species was stated by integer values. These helped to indicate the most effective medicinal plants for stomachaches.

*A paired comparison* was made for five medicinal plants used to treat snakebites in the study area. Seven key informants were selected to give rank to these medicinal plant species based on their efficiency in treating the disease (Table 2). This approach is useful in guiding decisions on which plants or plant parts to prioritize for further research in drug discovery.

*Direct matrix ranking* was carried out following the methods of Martine [1] and Cotton [17]. To compare the multipurpose use of a given species, six most widely utilized multipurpose plant species were selected out of the total medicinal plants, and seven use diversities of these plants were selected. The seven use values include medicinal, fodder, firewood, construction, charcoal, fencing, and income making. In the direct matrix ranking exercise, each key informant was asked to assign use values as follows: 5 = excellent, 4 = very good, 3 = good, 2 = less used, 1 = least used, and 0 = not used, for each species, and the average value of use diversity for a species was taken; the values for use reports across the selected species were summed up and ranked (Table 3). Similarly, six randomly selected key informants were selected for the ranking of the seven most threatened medicinal plants based on the report of their threatened condition by informants following Cotton [17] (Table 4). These helped to indicate the most threatened plant species. Accordingly, seven key informants were selected to assign use values to every six factors threatening medicinal plant species and asked to give a value of 1 to least destructive and 5 to most destructive ones(Table 5).

#### 2.2.5. Informant Consensus and Informant Consensus Factor

The informant consensus method was used to show certain plant species cited by informants against human ailment (Table 6), and the method was adopted from Alexiades [16]. The informant consensus factor (ICF) is calculated for each category to identify the agreements of the informants on the reported cures for the group of ailments (Table 7). The ICF was calculated as follows: the number of use citations in each category (nur) minus the number of species used (nt) divided by the number of use citations in each category minus one. The factor provides a range of 0 to 1, where a high value acts as a good indicator of a high rate of the informant consensus:

(1) $$\begin{array}{c} ICF=\frac{nur-nt}{nur-1}. \end{array}$$

## 3. Results and Discussion

### 3.1. Sociodemography of the Informants

A total of hundred informants were used for the study purposes; out of them, eighty (80%) male and twenty (20%) female informants took part in this study. Out of a hundred informants, forty-three (43%) of the informants were found between the ages of 20 and 40, and the remaining fifty-seven (57%) informants were older than 40 years old. Much of the knowledge of medicinal plants in the study area was obtained from older informants when compared with young people. Concerning the action taken when they faced a disease, out of the total informants, seventy (70%) of them responded to control by their own or self-care prepared from home remedies, twenty (25%) of informants visited the local traditional healer or herbalists, and the remaining five (5%) informants reported a modern clinic to be their first choice against the disease specifically for fibril illness, skin allergy, snakebite, jaundice, and scorpion bite. These results clearly show that most of the local people in the study area still now have their primary choice depending on traditional medicinal plants because based on the information gathered during the interview, the modern clinic has not given effective treatment for such kinds of diseases.

### 3.2. Source of Healing Knowledge

Most of the informants, especially the traditional medicine practitioners (TMP) or traditional healers, in the study area reported that the highest and the most commonly cited source of wisdom of healing was obtained informally from their family. Approximately 73% of the informants reported their family as the highest and most commonly cited source of wisdom for healing, followed by friends (15%). Additionally, a smaller percentage of informants mentioned that they gained knowledge through observation when others were practicing healing techniques (7%). doing (7%). Most of the traditional knowledge of medicinal plants is passed orally and secretly from generation to generation in fragile forms without any documentation. According to the information that was obtained from informants, still now some of the local traditional healers do not volunteer to transfer knowledge to their children, and also, due to modernization, most of the children (the young generation) showed reluctance and carelessness to gain knowledge from their family. Little emphasis has been given to ethnobotanical or traditional medicinal plants. These results agreed with the findings of the authors in [19], indicating that most sources of healing wisdom were obtained from the family. Other similar studies [20] show that most of the traditional knowledge of medicinal plants is passed orally and secretly along the family line from parents accounted for 73.6% followed by observation (19.4%) and learning (7%) from other people.

### 3.3. Taxonomic Diversity of Medicinal Plants in the Study Area

A total of 101 medicinal plant species belonging to 95 genera and 49 families were collected, identified, and documented across the study areas. The summary of the list of medicinal plant species used in Ensaro district to treat various disease conditions is given in Table 8. These medicinal plants are used to treat about 35 types of human ailments. Both families Fabaceae and Poaceae were represented by the highest number of medicinal plant species (9), followed by Asteraceae with 7 and Lamiaceae and Euphorbiaceae represented with 6 species. This result is in line with earlier studies conducted in Ethiopia that indicated Fabaceae had the largest number of medicinal plants [19, 21–23] reported that the family Fabaceae is the highest family. Other studies in a different part of Ethiopia such as the findings of [20, 24, 25] reported that the family Asteraceae is dominant followed by the family Lamiaceae.

### 3.4. Habitat of Medicinal Plants

**Figure 3:**
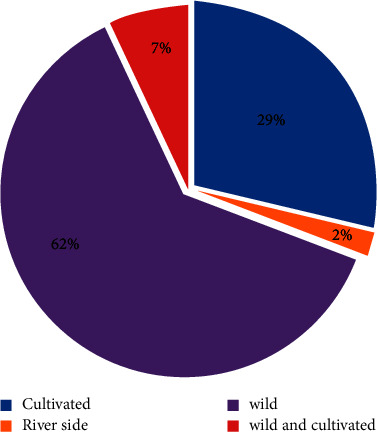
Sources and habitats of ethnomedicinal plant species in the study area.

In this study, a total of 101 plant species were collected and identified that were used as traditional medicine and harvested in the study area. Of the total medicinal plant, 62% was collected from the wild, followed by 29% from cultivated land, and the remaining 9% was collected from different habitats such as home gardens, on the side of the river, and in agricultural margins or fields (Figure 3). This result is in line with other studies [26] conducted in Ethiopia East Welega Zone of Oromia Regional State and [27] indicated that most medicinal plants are collected from the wild rather than home gardens.

### 3.5. Growth Forms of Medicinal Plants

**Figure 4:**
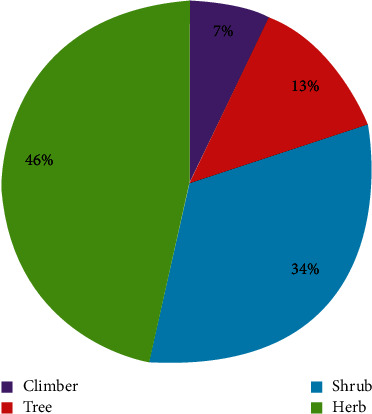
Proportional distributions of life forms of medicinal plants.

The growth form of medicinal plants in the study area showed that herbs are the dominant life form of medicinal plants. Among the reported medicinal plants, herbs consisted 47 (46%) followed by shrubs comprising 34 (34%), trees 13 (13%), and climbers 7 (7%) in the study area (Figure 4). Most of the different research studies conducted elsewhere in Ethiopia such as [19, 28, 29] and others reported that herbs constitute the highest category of medicinal plants. On the contrary, studies reported [24, 30–32] revealed that shrubs were the most used form of medicinal plants.

### 3.6. Conditions for Preparation of Herbal Remedies

**Figure 5:**
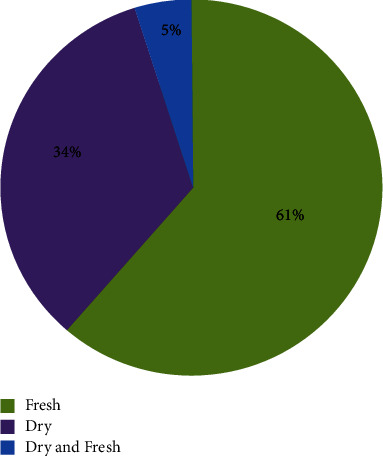
Condition of medicinal plants for remedy.

The local people of the study area reported that they prepared remedies using fresh, dried, or both types of plant materials. The result showed that the majority 64 (61%) of medicinal plants were prepared in fresh conditions, whereas 35 (34%) were prepared in dry conditions, and the remaining 5 (5%) were reported to be used in both dry and fresh forms (Figure 5). The reason why most of the respondents in the study area use fresh plant parts for the preparation of remedies could be due to the accessibility and availability of medicinal plants in the study area. This finding is consistent with the findings of [31, 33]. Moreover, other studies such as [22, 34] also reported that most prepared remedies were used immediately after harvest. However, people in the study area prepared medicine in the form of drying conditions to increase shelf life and for future long-time use because some medicinal plants are seasonal.

### 3.7. Route of Administration of Medicinal Plants and Way of Application

**Figure 6:**
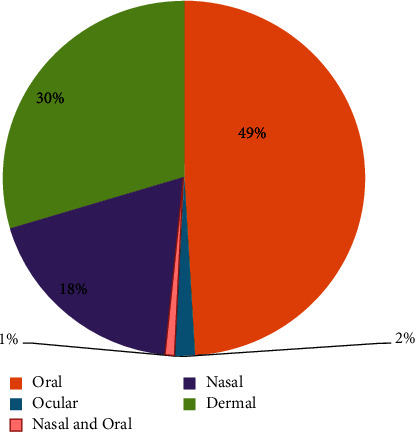
Route of administration of medicinal remedies.

Concerning modes of administration, medicinal plants were reported to be applied through different routes of administration based on the method of preparation, the actual site of alignments, and the type of disease treated. There are various routes of administration of traditional medicinal plants prepared products by the local community. The major routes of administration in the study area are oral, dermal, nasal, and optical. Oral administration is the dominant route (49%), followed by the dermal route (30%) and nasal route (18%) (Figure 6). These results are consistent with the findings of various ethnobotanical research studies in different areas of Ethiopia [26, 28].

### 3.8. Plant Parts Used

**Figure 7:**
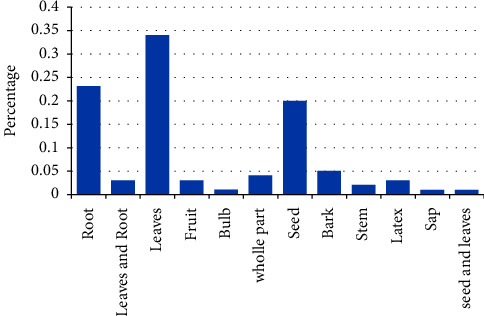
Proportion of plant parts used for the preparation of traditional medicine.

Different plant parts were used for medicinal purposes, and the leaf was the most frequently utilized part accounting (34%) for the remedy preparation, followed by roots (23%) (Figure 7). A higher preference for leaves over other plant parts could be due to ease of preparation and better treatment of a disease. While using leaves for medicinal preparations may not pose a significant threat to plant species, utilizing other parts such as roots, bark, and stems can have negative consequences. The overharvesting of these plant parts can disrupt the ecological balance and potentially endanger the survival of medicinal plant species. Therefore, it is crucial to implement proper conservation and sustainable measures to protect these plants and ensure their long-term viability. This finding is in line with the results of other ethnobotanical studies [31, 33] which reported that leaves were the most cited plant parts used in remedy preparations. However, other studies by Abebe and Ayehu [35] reported a different result which showed that roots were the most frequently utilized plant parts.

### 3.9. Method of Herbal Medicine Preparation

**Figure 8:**
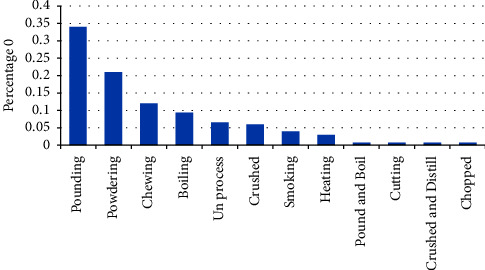
Method of herbal medicine preparation.

In the study area, medicinal plants have various methods of preparation and also the mode of application of the medicine for different types of ailments, and they have various preparation forms (Figure 8). Accordingly, the information gathered from the informants of the study site most frequently reported that the preparation method was pounding (34%), followed by grinding (powdering) (21%) and chewing (12%).

### 3.10. Importance of Medicinal Plants in the Study Area

#### 3.10.1. Preference Ranking

When there were different species reported for the same health problem, people show a preference for one over the other, depending on the potential of plants to treat the disease. Accordingly, preference ranking analysis was conducted on nine medicinal plants that were reported for treating stomachaches. As shown in Table 1, the result of this analysis revealed that preference ranking of the nine most common and widely used medicinal plants that were reported to treat stomachaches (because it is a frequently occurring disease in the study site) indicated that *Cucumis ficifolius* is the first one, followed by *Ziziphus spina-christi*, and *Maytenus arbutifolia*, *Ruta chalepensis*, *Foeniculum vulgare*, *Zingiber officinale*, and *Calpurnia aurea* have ranking from 3^rd^ to 7^th^, respectively, which are preferred for the treatment of stomachaches.

#### 3.10.2. Paired Comparison

Seven key informants performed the paired comparison of five medicinal plants, and the value is summed, summarized, and finally ranked (Table 2). It was found that *Andrachne aspera* species ranks first for the treatment of snake poisons followed by *Cyphostemma cyphopetalum* which ranks 2^nd^. The remaining *Clerodendrum myricoides*, *Carissa spinarum*, and *Barleria priorities* were 3^rd^, 4^th^, and 5^th^ ranks, respectively. *Carissa spinarum* and *Barleria priorities* were the least preferred species to treat the snakebite disease in the area. The rank shows the efficacy of the plant to treat snake poisons according to a long practice of local people using plants to treat these ailments.

#### 3.10.3. Direct Matrix Ranking

Several medicinal plants were reported for having to be multipurpose species which are being utilized for a variety of uses due to their medicinal value (Table 3). Six commonly reported multipurpose species and seven use categories were involved in direct matrix ranking with five key informants. The results of the direct matrix ranking revealed that *Eucalyptus globulus* and *Ziziphus spina-christi* were ranked 1^st^ and 2^nd^ and hence are the most preferred medicinal plants by local people for various uses. Due to this reason, *Ziziphus spina-christi* is a threatened species in the study site, but *Eucalyptus globulus* is abundant because it is planted by humans.

#### 3.10.4. Informant Consensus

Concerning informant consensus analysis of informants in the study area showed that some medicinal plants were more popular or cited by many individuals than others (Table 6). However, certain medicinal plant species were independently cited by many of the informants for their medicinal uses against human ailments. Accordingly, *Ocimum lamiifolium* was the most cited traditional medicinal plant in the study area accounting 75 (75%) followed by *Cucumis ficifolius* 41 (41%) in the study area.

#### 3.10.5. Informant Consensus Factor

Based on the findings of this study, the ICF of medicinal plant usage was found between the ranges of 0.75 and 0.97 per illness category (Table 7). As a result, it was found that febrile illness (headache, “Mitch”) scored the highest informant consensus factor of 0.97 followed by stomachaches having a score of an ICF of 0.95. This result indicates that the informants use relatively few species to manage specific disease conditions as well as have consistency in the use of plant species, while a low value indicates that the informants disagree on the species to be used in the treatment within a category of illness.

#### 3.10.6. Dosage Use, a Side Effect of Herbal Medicine, and Its Antidotes

The majority of traditional healers use different measurements for dosage prescription and apply them to treat different health problems and have not seen their clear side effects. Traditional medicine, such as any other form of medication, can also have potential side effects. Some common side effects of traditional medicine may include vomiting, nausea, diarrhea, gastric (burning sensation), mouth smell, loss of appetite, sweating, and urination which were the most common side effects for those remedies taken orally. Similar side effects of medicinal plants were also reported in a research paper [36]. But communities use different local materials (units) for the measurement of dosage and the duration of administration of herbal medicine. Local units, for instance, glass, half cup, full cup, one or two spoons, finger length for bark, number of the root, and stem, were employed. Several different parts of plants such as leaves, seeds, fruits, and shoot tips were also used to estimate and fix the amount dosage. For example, seven seeds of *Lens culinaris* were used for the treatment of skin disease caused by a spider. Seven fresh leaves of *Carissa spinarum* are chewed to treat snake poison, and seven *Capsicum annum* fruits to prepare herbal medicine for the treatment of jaundice are used. The full-dose determination varied from a healer to a healer, and the dose given depends on age, physical strength, and health conditions. For example, a half cup was used for children, and a full cup was used for an adult. Similar findings were also reported using different local units to determine the dosage of herbal medicine [19].

Different antidotes are administered by the local healer against vomiting, diarrhea, and gastric burning such as porridge of lentil, drunk filtered (liquid) of boiled lentil, milk, coffee, “atemit,” red teff porridge, and the mixture of coffee and milk. In addition to these, some foods such as bread and fish are not recommended to consume for some disease. This finding is similar to that reported by another researcher [37] who reported milk, coffee, and red teff porridge were used as antidotes for different herbal medicines.

#### 3.10.7. Threats to Medicinal Plants and Conservation Practices

People of the study area reported that ten and fifteen years ago in most parts of Ensaro woreda and its surroundings, the accessibility of plant species for medicinal purposes, construction, and firewood was very high both in the number and diversity and can access near home. Currently, it is too difficult to get these traditional medicinal plants easily. Due to population growth, the demand for wood material, agricultural expansion, and urbanization are increased and thus have effects in threatening the medicinal plants and associated indigenous knowledge of the study area. Different threat factors were mentioned by the informants. The major factors arise mostly from anthropogenic causes. Among the problems, firewood collection due to high population pressure (1st rank) was considered the main threat to medicinal plants, and others are charcoal production, fencing, and agricultural expansion which were ranked 2^nd^, 3^rd^, and 4^th^, respectively (Table 5).

A similar study [25] reported that medicinal plants used by Minjar Shekora people in north Shewa Ethiopia were highly threatened by deforestation for agricultural expansion and other purposes. Concerning the conservation of traditional medicinal plants and associated indigenous knowledge, there were no successfully available conservation efforts in the woreda. But, since two or three years ago, the woreda had started to set special rules concerning natural resource protection mechanisms, some of which strictly forbid cutting any tree from anywhere, organizing the farmer to construct terraces by selecting the area which is more exposed to erosion, and the woreda agricultural office distribute indigenous tree nursery to the different agroecological zones of kebeles for plantation. That is very important to save and recover degraded land. On the other hand, it was also observed that local farmers make use of their indigenous knowledge in protecting important plant species on their farmlands, home gardens, and as life fences. In addition to this, some traditional healers try to cultivate very few species in their home gardens that cannot easily be found within the area at any time. Still, now, the woreda administration is not giving priority to conserving traditional medicinal plants and their associated indigenous knowledge of the people, rather than the whole biodiversity conservation practices.

#### 3.10.8. Threatened Medicinal Plants

According to key informants, it is revealed that medicinal plant species were considered to be threatened in the study area (Table 4). Based on the perception of the community, *Withania somnifera* is the most threatened plant species followed by *Andrachne aspera*, whereas *Cucumis ficifolius*, *Olea europaea*, and *Ximenia americana* have taken 3^rd^, 4^th^, and 5^th^ ranks, respectively.

#### 3.10.9. Marketability of Medicinal Plants in Ensaro Woreda

The majority of traditional medicinal plants were not available for sale in the local markets of Ensaro subcity, and also, medicinal plants in the study area are not sold in the market for the specific purpose of medicine. The list of medicinal plants that were traded in the study area is given in Table 9. Even if local people prefer either collecting these plants by themselves from the available areas (vegetation) in the district to prepare the medicines or prefer going directly to local healers to obtain treatments instead of buying medicinal plants from the market. However, medicinal plant species that are widely traded that serve different purposes such as spicing foods, firewood, and construction are some of the most important and sold in the market.

## 4. Conclusion

Ensaro district is relatively rich in medicinal plants' diversity and associated indigenous knowledge. The knowledge of plant uses for various purposes also varies among various social groups. Large numbers of medicinal plant species were collected from the wild, whereas the remaining ones were collected from cultivated land. Therefore, awareness creation is the time needed to improve the local community's knowledge of the importance and management of plants both in their natural habitats (in situ) and out of their natural habitats (ex situ) such as field gene banks and home gardens. The government also gives recognition to the local healer to apply their traditional practices which are known for their proven safety and effectiveness to avoid erosion of the indigenous knowledge and to ensure its sustainable use. Most of the plants are found under threats in the study area, which is directly related to the decline of traditional medicinal knowledge. One of the main reasons for the decline of these traditional medicinal plants in the area arises from deforestation for firewood, charcoal, agricultural expansion, and construction. In addition to these threats, the utilization of these plants for medicinal purposes is negligible. Other threats to traditional indigenous knowledge are the way of transferring knowledge which is oral-based, the reluctance of the young generation to gain knowledge, the expansion of modern health institutions, the influence of modern education, and awareness factors. Therefore, besides conserving such a wealth of information hidden among the local people, it is also important to connect and apply them to modern knowledge of science and technology to meet the ever-increasing requirements of humankind. Therefore, it is important to create awareness about the conservation of these biological resources, and the importance of maintaining the knowledge of herbal medicine should be made among the healers to avoid erosion of indigenous knowledge and to ensure its sustainable use.

## Figures and Tables

**Table 1:** Preference ranking of medicinal plants used to treat stomachaches.

Medicinal plant species	Respondent							
	R1	R2	R3	R4	R5	R6	Total	Rank
*Foeniculum vulgare*	6	5	4	5	3	5	28	5^th^
*Calpurnia aurea*	3	4	1	1	4	2	15	7^th^
*Cymbopogon citratus*	1	2	4	2	1	1	11	9^th^
*Ziziphus spina-christi*	8	8	8	9	8	6	47	2^nd^
*Ruta chalepensis*	5	7	5	7	6	8	38	4^th^
*Cucumis ficifolius*	9	9	9	8	9	9	53	1^st^
*Salvia nilotica*	2	1	2	4	2	3	14	8^th^
*Zingiber officinale*	4	3	3	3	5	4	23	6^th^
*Maytenus arbutifolia*	7	6	7	6	7	7	40	3^rd^

**Table 2:** A paired comparison of medicinal plant species used to treat snakebite (poisons).

Medicinal plants	Informants labelled								
	I1	I2	I3	I4	I5	I6	I7	Total	Rank
*Cyphostemma cyphopetalum*	1	2	2	1	4	3	2	15	2^nd^
*Carissa spinarum*	2	1	2	3	1	1	1	11	4^th^
*Barleria prionitis*	2	2	2	1	1	1	1	10	5^th^
*Clerodendrum myricoides*	1	2	1	2	2	2	3	13	3^rd^
*Andrachne aspera*	4	3	3	3	2	3	3	21	1^st^

**Table 3:** Direct matrix ranking of six multipurpose plant species by five informants based on seven use criteria.

Plant species	Use categories							Total score	Rank
	Firewood	Construction	Fodder	Charcoal	Medicinal	Fencing	Income		
*Croton macrostachyus*	4	3	3	3	4	3	3	23	5^th^
*Cordia africana*	4	4	3	3	3	4	5	26	3^rd^
*Vernonia amygdalina*	3	2	3	1	4	3	2	18	6^th^
*Eucalyptus globulus*	3	5	2	4	4	5	5	28	1^st^
*Ziziphus spina-christi*	3	5	4	4	4	4	3	27	2^nd^
*Olea europaea*	4	4	3	3	4	4	3	25	4^th^

5 = best; 4 = very good; 3 = good; 2 = less used; 1 = least used; 0 = no value.

**Table 4:** Ranking of threatened medicinal plant species in the study area.

Medicinal plant species	R1	R2	R3	R4	R5	R6	Total	Rank
*Withania somnifera*	4	3	4	4	3	4	22	1^st^
*Olea europaea*	3	3	2	2	3	3	16	4^th^
*Ximenia americana*	3	2	2	3	2	3	15	5^th^
*Cucumis ficifolius*	3	3	4	3	3	4	20	3^rd^
*Calpurnia aurea*	2	2	3	2	3	1	13	7^th^
*Andrachne aspera*	4	3	4	3	3	4	21	2^nd^
*Ziziphus spina-christi*	2	2	2	3	2	3	14	6^th^

**Table 5:** Direct matrix ranking results of factors perceived as threats to medicinal plants.

Threaten factor	Respondents								
	R1	R2	R3	R4	R5	R6	R7	Total	Rank
Agricultural expansion	2	3	3	3	3	3	3	20	4^th^
Deforestation for firewood	5	6	6	6	6	5	6	40	1^st^
Charcoal	6	5	5	4	5	6	4	35	2^nd^
Construction	3	2	1	2	2	1	2	13	5^th^
Drought	1	1	2	1	1	2	1	9	6^th^
Fence	4	4	4	5	4	4	5	30	3^rd^

**Table 6:** Result of the informant consensus on medicinal plants in the study area.

No	Scientific name	Local name	No. of informant cited	%
1	*Ocimum lamiifolium* Hochst. ex Benth	Damakese	75	75
2	*Cucumis ficifolius* A. Rich	Ywmder ebuye	41	41
3	*Eucalyptus globulus* Labill	Nech beharzafe	39	39
4	*Zehneria scabra* (Linn.f.) Sond.	Shehara kitel	36	36
5	*Allium sativum* L	Nech shekuret	35	35
6	*Croton macrostachyus* Hochst	Bisana	27	27
7	*Lepidium sativum* L	Feto	23	23
8	*Ruta chalepensis* L	Tenadam	22	22
9	*Premna schimperi* Engl	Chocho	20	20
10	*Salvia nilotica* Juss. ex Jacq	Hulegeb	20	20
11	*Zingiber officinale* Rosco	Zengebel	19	19
12	*Vicia faba* L	Bakila	19	19
13	*Nicotiana tabaccum* L	Tebaho	18	18
14	*Ziziphus spina-christi*	Geba	18	18
15	*Cyphostemma cyphopetalum* (Fresen.)	Gendosh	18	18
16	*Andrachne aspera*	Etse tekeza	17	17
17	*Echinops kebericho* Mesfin	Keberecho	14	14

**Table 7:** Informant consensus factor by categories of diseases in the study area.

Diseases category	Number of plant species used	Number of use citations (*N*_ur_)	ICF
Dermatological disease (skin rash and skin allergy (caused by spider-like insects)	8	87	0.91
Gastrointestinal disorder (diarrhea, gastric, and constipation)	11	96	0.89
Body swelling (begunig) and wound	8	91	0.92
Febrile illness (headache, “mitch”)	5	148	0.97
Jaundice	5	40	0.89
Stomachaches	6	118	0.95
Respiratory disease (tonsillitis, common cold)	11	68	0.85
Organ disease, eye disease, teeth, and ear	10	91	0.9
Internal parasite ascaris and tapeworm	4	13	0.75
Malaria	4	54	0.94
Snakebite	7	90	0.93

**Table 8:** List of medicinal plants used to treat human ailments in the study area.

Scientific name	Local name	Family	Habit	Habitat	Part used	Co	Preparation	Mode of application	Method of preparation	Route	Disease treated
*Allium cepa* L.	Keye shekuret	Liliaceae or Alliaceae	H	C	Bu	F	Roast the mixture of chopped onion with egg and oil without adding water. Finally, add salt, then eat with injera, used to treat diarrhea	Eating	Chopping	Oral	Diarrheal
*Acacia abyssinica* Hochst. Ex Benth.	Girare	Fabaceae	T	W and C	L	F	Pound the mixture of seven leaves of *Acacia abyssinica* and seven leaves of *Rhamnus prinoides*, soak in a bitter calabash container with water, and finally immerse the baby	Washing	Pounding	Dermal	Undersized baby (yelijoch mkechech)
*Cymbopogon citratus* (DC. ex)	Tejesar	Poaceae	H	C	Wp	F	Pound the whole plant parts, mix with water, and then drink it Crushed and mixed with water and then drink it	Drinking Drinking	Pounding Crushing	Oral Oral	Stomachache Mogn bagegn (high blood pressure)
*Kalanchoe Schimperiana* A. Rich.	Endahahula	Crassulaceae	H	W	L	F	The leaf was heated and then attached to the wound	Put on	Heating	Dermal	Wound
*Leonotis nepetifolia* (L.) R. Br.	Raskimire	Lamiaceae	H	W	R	D	Dried the root and fumigated	Fumigating	Smoking	Nasal	Mitch (disease caused by excess sun is believed to cause mitch “sunstroke”)
*Coffea arabica* L	Buna	Rubiaceae	S	C	S and L	F	Boil the mixture of powdered coffee with butter and then drink it	Drinking	Powdering	Oral	Cough
*Hordeum vulgare* L	Gebese	Poaceae	H	C	S	D	Roasted seed ground and mix with lemon and wait until fermented and then drink it	Drinking	Powdering	Oral	Diarrheal
*Lippia adoensis var adoensis*	Kessie	Verbenaceae	H	C	R	D and F	Chewing the root and then swallow	Swallowing	Chewing	Oral	Smallpox
*Tephrosia bracteolata*'Guill. and Perr.	Gerngera	Fabaceae	S	W	R	F	Chewing the root and swallow	Swallowing	Chewing	Oral	Snakebite
*Persea americana* Mill	Avocado	Lauraceae	T	C	L	F	The leaves were boiled with water and drink the liquid portion after filtration	Drinking	Boiling	Oral	Kidney infection
*Ocimum lamiifolium* Hochst ex Benth	Demakese	Lamiaceae	S	W	L	F	The leaves were boiled with water and put in very hot metal in the boiled mixture and finally fumigated Pound the leaves and squeeze them, then mix them with honey and finally drink it	Fumigating Drinking	Boiling Pounding	Nasal Oral	Mitch Mitch
*Saccharum officinarum* L.	Shenkora ageda	Poaceae	H	C	Stem	F	Crushed the stem of a plant and boiled finally drinking it	Drinking	Crushing	Oral	Cough and common cold
*Schinus molle* L.	Kunedo berebere	Anacardiaceae	T	C	S	D	Powdered the dried seed and drank it	Drinking	Powdering	Oral	Stomachache
*Carica papaya* L	Papaya	Caricaceae	T	C	F	D and F	Eating the fruit every morning Crushed and dried the leaves and then powdered and applied to the wound	Eating Put on	Unprocessed Powdering	Oral Dermal	Gastric Wound
*Carthamus tinctorius*	Yabesha suff	Asteraceae	H	C	S	F	Boil the mixture of *Sesamum indicum, Carthamus tinctorius,* and *Guizotia abyssinica* seeds and drink it	Drinking	Boiling	Oral	Cough
*Catha edulis* (Vahl) Forssk. ex Endl.	Chate	Celastraceae	S	C	L	F	Pound the leaves and boil and drink them at night for 3 to 4 consecutive days	Drinking	Pounding and boiling	Oral	Cough
*Foeniculum vulgare* Miller	Ensilale	Apiaceae	H	W	Wp	F	Pounded the leaves and stem and boiled with water and drank Chewing the plant and mixing it with salt, applied to the affected part	Drinking Holding	Pounding Chewing	Oral Oral	Kidney and stomach ache Teeth
*Nigella sativa* L.	Tekur azemude	Ranunculaceae	H	C	S	D	Crushed the seed and sniff Keep the seed in ventilated cloth and then inhaled it	Sniffing Sniffing	Crushing Unprocessed	Nasal Nasal	Cough Common cold
*Olea europaea* L. ssp. *Cuspidata* (Wall. ex G. Don) Cif.	Woyera	Oleaceae	T	C and W	Stem	D & F	The wood of *Olea europaea* is inserted into a pitcher and is given high heat and distilled. Then applied the distilled droplets to the affected part	Dropping	Crushing and distilling	Dermal	Wound
*Millettia ferruginea* (Hochst.) Bak.	Berebera	Fabaceae	T	W	R	D	Powdered the deride root and drank it	Drinking	Powdering	Oral	Impotency
*Musa* x *paradisiaca* L.	Muse	Musaceae	H	C	F	F	Rubbed affected parts with the peel of the banana	Rubbing	Unprocessed	Dermal	Cheffa or eczemas
*Cucurbita* spp	Gim hareg	Cucurbitaceae	C	W	R	F	Inhaling after crushing the root	Sniffing	Crushing	Nasal	Malaria
*Withania somnifera* (L.) Dunal in Dc.	Gezwa	Solanaceae	S	W	L	D	Crushed and powdered the roots of *Withania somnifera*, *Carissa spinarum*, *Verbena officinalis*, *Capparis tomentosa*, *Cucumis ficifolius*, and whole parts of *Artemisia afra*, *Ruta chalepensis*, Bulb of *Allium sativum* then fumigated	Fumigating	Pounding	Nasal	Evil eye
*Clerodendrum myricoides* (Hochst.) Vatke	Misirch	Lamiaceae	S	W	Ba	F	In the early morning, the local healer before meeting with other people collects the bark of a plant with a mouth and then drops the bark to the mouth of a snake-bitten person. The healer never talked during this process	Swallowing	Unprocessed	Oral	Snakebite
*Artemisia afra* Jacq. Ex Wild.	Chekugn	Asteraceae	H	W	Hp	F	Inhaled the smash leaves and put them in your pocket Smashed the leaves and drank	Sniffing Drinking	Pounding Pounding	Nasal Oral	Evil eye and common cold Acute cough
*Guizotia abyssinica* Cass	Nug	Asteraceae	H	C	S	D	Boiled the seed with water and then drank it after filtration Boiled the mixture of *Guizotia abyssinica* seed, tenadam, and areka and drank it	Drinking Drinking	Boiling Boiling	Oral Oral	Cough Common cold
*Cicer arietinum* L.	Shenebera	Fabaceae	H	C	S	D	Bake the powder of cicer as injera and eat Mix the powder of the cicer with water and drink Boil the chickpea seed (mangregeb) and mix with salt and eat	Eating Drinking Eating	Powdering Powdering Boiling	Oral Oral Oral	Malaria Malaria To heal the broken bone
*Epilobium hirsutum* L.	Woneze ademek	Onagraceae	H	W (river)	L	F	Crushed the mixture of *Epilobium hirsutum* leaf, seven capsicum fruit, a bar of salt, Shiro, and finally ate with one-third of injera	Eating	Pounding	Oral	Jaundice
*Rhus retinorrhoea* Krauss	Tlem	Anacardiaceae	S	W	L and R	F	Pound the leaf and root, then mix with water, and finally drink it	Drinking	Pounding	Oral	Liver
*Maytenus arbutifolia* (A. Rich.) Wilczek	Tekur Atate	Celastraceae	S	W	L	F	Chew the leaf and swallow	Swallowing	Chewing	Oral	Stomachache
*Ziziphus spina-christi*	Geba (Qurqura)	Rhamnaceae	S	W	Ba	F	Chew the bark and swallow	Swallowing	Chewing	Oral	Stomachache
*Vicia faba* L.	Bakela	Fabaceae	H	C	S	D	Chew the seed and tie on to the wound Soaked the seed in water and roasted it finally, consuming it after adding salt	Put on Eating	Chewing Boiling	Dermal Oral	Wound Diarrhea
*Trticum* spp	Sinde	Poaceae	H	C	S	D	Chew the seed and tie on to the wound	Put on	Chewing	Dermal	Wound
*Croton macrostachyus* Hochst	Bisana	Euphorbiaceae	T	W	L	F	Boiled the mixture of bisana, nech beharzaf, and damakese together finally fumigated	Fumigating	Boiling	Nasal	Mitch
*Barleria prionitis*	Yesete melase	Acanthaceae	S	W	L	F	Chew the bark and swallow Pound the leaves and apply to the affected parts	Swallowing Put on	Chewing Pounding	Oral Ocular	Snakebite as first aid Eye disease
*Moringa stenopetala*	Morinega	Moringaceae	T	W	L	F	Pound the leaves and drink them	Drinking	Pounding	Oral	Blood pressure
*Melia azedarach* L.	Nim	Meliaceae	T	C	Ba	F	Pound the bark and drink it after mixing it with water	Drinking	Pounding	Oral	Malaria
*Phytolacca dodecandra* L'Herit	Endode	Phytolaccaceae	S	W	R	F	Pound the fresh root and mix it with water and then drink it	Drinking	Pounding	Oral	Abortion and malaria
*Euphorbia tirucalli* L	Kenchib	Euphorbiaceae	S	C	Sap	F	After the scorpion bite, snicks the bitten-boy part with a blade and applies the sap of *Euphorbia tirucalli* to the snicked part	Dropping	Unprocessed	Dermal	Scorpion bite
*Rumex nepalensis* Spreng.	Tulet	Polygonaceae	H	W	R	F	Chew the root and swallow Pound the root and tied on the wound	Swallowing Put on	Chewing Pounding	Oral Dermal	Stomachache Wound
*Acokanthera schimperi* (A. DC.) Schweinf.	Mereneze	Apocynaceae	S	W	R	D	Dried and powdered the root and fumigated Crushed the root and mixed with water and then drank it	Fumigating Drinking	Powdering Crushing	Nasal Oral	Evil eye Vomiting
*Datura stramonium* L.	Asetnager	Solanaceae	H	W	L	F	Crushed the leaves and applied them on the head Pound the leaves and squeeze and then apply them to the affected body part	Painting Painting	Pounding Pounding	Dermal Dermal	Dandruff and wound Wound
*Daucus carota* L.	Karrote	Apiaceae	H	C	R	F	Chewing the root and swallowing it	Swallowing	Chewing	Oral	Kidney and eye clearance
*Dovyalis abyssinica* (A. Rich.) Warb.	Koshim	Salicaceae	S	W and C	F	F	Heated the half of fruit and then tied it to the affected part	Put on	Heating	Dermal	Hemorrhoids
*Embelia schimperi* Vatke	Enkoko	Myrsinaceae	S	W	L	F	Pounding the seed of (*Embelia schimperi*) and Nug (*Guizotia abyssinica*) and eating with Kita	Eating	Pounding	Oral	Tapeworms
*Prunus africana*	Tekur echete	Rosaceae	S	W	L	D	Powdered the dried leaves and then smoked or fumigated	Fumigating	Powdering	Nasal	Evil eye
*Clutia lanceolata* Forssk	Feyele feg	Euphorbiaceae	S	W	R	D	Powdered the dried root and then smoked or fumigated	Fumigating	Powdering	Nasal	Evil eye
*Coria africa* Lam	Waneza	Boraginaceae	T	W and C	L	F	Pound the leaves and boil with water and finally fumigated	Fumigating	Pounding and boiling	Nasal	Cough and mitch
*Ferula communis* L.	Dog	Apiaceae	H	W	L	F	The leaves crushed and pounded and then inhaled it	Sniffing	Pounding	Nasal	Evil eye
*Rosa abyssinica* Lindley	Kega	Rosaceae	S	W	R	D	Burn the dried root and fumigated	Fumigating	Smoking	Nasal	Evil eye
*Myrtus communis* L.	Ades	Myrtaceae	S	W	L	D	The powder of dried leaf mixed with butter (paste) Mixed the dried leaf powder with butter	Painting Painting	Powdering Powdering	Dermal Dermal	Dandruff Itch
*Aloe pulcherrima* Gilbert and sebsebe	Sete eret	Aloaceae	H	W	Latex	D and F	Cut the leaves and attach them to the affected part or powder of dried leaves and apply on the affected parts	Painting	Powdering	Dermal	Begunji and wound
*Aloe debrana* Christian	Wode eret	Aloaceae	H	W	Latex	F	Cut the leaves and attach them to the affected part	Put on	Unprocessed	Dermal	Wound
*Lycopersicon esculentum* Mill	Timatim	Solanaceae	H	C	L	F	Pound the mixture of leaves of *Croton macrostachyus, Clerodendrum myricoides*, *Rhamnus prinoides,* and *Zehneria scabra* were applied on the affected body part	Painting	Pounding	Dermal	Yegedegedewa
*Silene macrosolen* A. Rich.	Wogeret	Caryophyllaceae	H	W	L and R	F	Chewing the root and swallowing or pounding the leaves and drink	Drinking	Chewing	Oral	Tapeworms and headache
*Trigonella foenumgraecum* L	Abish	Fabaceae	H	C	S	D	Dried the boiled seed and ground it, then mixed it with water, and finally drank it after filtration	Drinking	Boiling	Oral	Constipation
*Zehneria scabra* (Linn.f.) Sond.	(Hareg resa)	Cucurbitaceae	C	W	L	F	Pounded the leaves and squeezed and then applied to affected body parts	Painting	Pounding	Dermal	Yegedegedewa
*Inula confertiflora* A. Rich.	Woyenagift	Asteraceae	S	W	L	F	Crushed or chewed the leaves and powdered and then applied to the affected eye	Dropping	Powdering	Ocular	Eye disease
*Lagenaria siceraria* (Molina) Standl	Qil	Cucurbitaceae	C	C & W	S	D & F	Mix the seed with the powder of teff and eat Rubbed by the fleshy part of the fruit	Eating Rubbing	Powdering Unprocessed	Oral Dermal	Rabies Dandruff
*Cyphostemma adenocaule* (Steud. ex A. Rich.)	Aserkush	Vitaceae	C	W	L	F	Crushed the leaves and tied them to the affected body part	Put on	Pounding	Dermal	Wound
*Hagenia abyssinica* (Bruce) J. F. Gmel	Koso	Rosaceae	T	W and C	S	F	Fruits are ground and mixed with milk and drunk in the morning empty stomach	Drinking	Pounding	Oral	To treat tapeworm
*Gossypium barbadense* L.	Tite	Malvaceae	H	C	S	D	Burn dried seed at home	Sniffing	Smoking	Nasal	To repel snake
*Eragrostis tef* (Zucc.)	Keye Teff	Poaceae	H	C	S	D D D	The seed is ground by a local or traditional grinder and then baked as injera and eaten Prepare gruel (atemit) with a mixture of teff and barley flour and drink it Prepare the dough, filter the sour part of the dough, and then drink it	Eating Drinking Drinking	Powdering Powdering Powdering	Oral Oral Oral	Acute diarrhea Gastritis Snakebite
*Asparagus africanus* Lam.	Serti	Asparagaceae	C	W	L	F	The leaves are mixed with *Zehneria scabra* leaves and pounded and applied on the affected parts	Painting	Pounding	Dermal	Yegedegedewa
*Securidaca longipedunculata* Fresen	Etse menahi	Polygalaceae	H	W	R	F	Chew the root and swallow	Swallowing	Chewing	Oral	Evil eye
*Clematis hirsuta* Perr.	Azohareg	Ranunculaceae	C	W	L	F	Pounded the leaves and tied them to the affected part	Painting	Pounding	Dermal	Yegedegedewa
*Jasminum abyssinicum* Hochest. Ex DC.	Tenbelele	Oleaceae	S	W	L	F	Pounded the leaves and drank	Drinking	Pounding	Oral	Koso and ascaris
*Sorghum* spp	Zegada	Poaceae	H	C	S	D	Roasted the seed and ground, the powder mixed with water and boiled, and finally drunk	Drinking	Powdering	Oral	Constipation
*Sesamum indicum*	Selit	Pedaliaceae	H	C	S	D	Pound the roasted seed and boil and then drink Pound the mixture of roasted *Sesamum indicum* and *Trigonella foenum-graecum* seed and then apply on the affected parts	Drinking Put on	Boiling Pounding	Oral Dermal	Common cold Bone breakage
*Glinus lotoides* L. var lotoides	Metere	Molluginaceae	C	W	Ba	D	Powdered the dried bark and mixed with the roasted seed of *Guizotia abyssinica*, and finally eaten with Kita (thin bread)	Eating	Powdering	Oral	Koso
*Euphorbia abyssinica* J. F. Gmel.	Qulqual	Euphorbiaceae	T	W	Latex	F	Heated the latex and bark together and tied them to the affected parts	Put on	Heating	Dermal	Kintarote
*Eleusine floccifolia*	Akirma	Poaceae	H	W	S	F	Chewing the flower and seed together and then applying on the affected part	Painting	Chewing	Dermal	When snake looks (ebach)
*Otostegia integrifolia*	Tenjute	Lamiaceae	S	C	Wp	D	Powdered the dried leaves and then fumigated. Sometimes mixed with other plants Pound the leaves and drink	Fumigating Drinking	Powdering Pounding	Nasal Oral	Mitch Ascarid
*Becium grandiflorum* (Lam.) hirsuta Perr.	Matosh	Lamiaceae	S	W	L	F	Pound the leaves and attached them to the affected part	Put on	Pounding	Dermal	Yegedegedewa (spider disease)
*Justicia schimperiana* (Hochst. ex Nees) T. Anders.	Sensel	Acanthaceae	S	Roadside	L and R	F	Pound leaves and root and drank The leaves are mixed with *Croton macrostachyus* and pounded and then drunk with a small amount of one cup unless it kills	Drinking Drinking	Pounding Pounding	Oral Oral	Malaria, diarrhea, and headache Jaundice
*Brucea antidysenterica* J. F.	Chefa kitel	Simaroubaceae	S	W	S	F	Pound the seed together with the leaves of fafugn and then rub them on the affected part for 3 to 4 days with a less amount because it is highly irritated	Rubbing	Pounding	Dermal	Body rash
*Plantago lanceolata* L.	Yekura wosefa	Plantaginaceae	H	W	L	F	Fresh leaves crushed and attached to affected parts	Put on	Pounding	Dermal	Wound
*Berassica nigera*	Senafich	Brassicaceae	H	Cu	S	D	Boiled the powder and then mixed with lemon and germinated *Vicia faba*	Eating	Powdering	Oral	Common cold
*Albizia anthelmintica* Brongn.	Musena	Fabaceae	S	W	Ba	D	The bark is dried and powdered, then mixed with roasted *Guizotia abyssinica*, and finally eaten with Kita (thin bread)	Eating	Powdering	Oral	Tapeworm Taeniasis
*Gomphocarpus purpurascens* A. Rich.	Tefrindo	Asclepiadaceae	H	W	R	D	The root is chopped and dried (the plant is not allowed to enter into house) Then mix with clean teff (keye teff) , grind the mixture with a traditional grinder, bake it like injera, cut the one injera in seven equal parts and roll all individual, and give to all seven rolled injera to eat for a eight months pregnant female	Eating	Powdering	Oral	Shotelaye
*Laggera crispata* (Vahl) Hepper & Wood	Alashume	Asteraceae	S	W	L	F	Chopped the leaves and pounded and drank it	Drinking	Pounding	Oral	Stomachache
*Laggera tomentosa*	Chese nedede	Asteraceae	H	W	L	F	Chopped the leaves and pounded and drank it	Drinking	Pounding	Oral	Ascaris
*Andrachne aspera*	Etse tekeza	Euphorbiaceae	H	W	R	D	Powdered the dried root and fumigated it	Fumigating	Powdering	Nasal	Evil eye
*Osyris quadripartita* Dec.	Kert	Santslaceae	S	W	R	D	Dried and powdered and then smoked it	Fumigating	Smoking	Nasal	Evil eye
*Solanum nigrum* L.	Tekur awute	Solanaceae	H	W	L	F	Crushed and tied at the problematic part	Put on	Pounding	Dermal	Insect allergy
*Dodonaea angustifolia* L. f.	Kitikita	Sapindaceae	S	W	L	F	Burn or heat the leaves and then attach them to the affected body part	Put on	Heating	Dermal	A wound caused by fire burn
*Polygala rupicola* A.	Etselebona	Polygalaceae	S	W	R	F	Chewing the root and swallowing the liquid part	Swallowing	Chewing	Oral	Snakebite
*Sida Schimperiana* Hochst. A. Rich.	Chefereg	Malvaceae	S	W	L	D	Powdered the crushed root and then sniffed after burning	Fumigating	Smoking	Nasal	Evil eye
*Artemisia absinthium* L.	Areti	Asteraceae	H	W	L	F	The roots were crushed and powdered and then sniffed	Fumigating	Pounding	Nasal	Evil eye
*Thalictrum rhynchocarpum* Dill. Quart.-Dill & A. Rich	Serabizu	Ranunculaceae	S	W	R	D	The roots were crushed and powdered and then sniffed	Fumigating	Rushing	Nasal	Evil eye
*Pennisetum sphacelatum*' (Nees) Th. Dur. and Schinz	Sededo	Poaceae	H	W	R	D	The root of *Verbascum sinaiticum*, *Aloe pulcherrima,* and *Eleusine floccifolia* is cut into three equal parts and then keep all in the skin of a goat and hang on the neck	Put on	Cutting	Dermal	Shotelaye
*Tragia cinerea* (Fax.)	Alleblabit	Euphorbiaceae	H	W	R	F	Chewing the root and swallowing the liquid and putting in teeth	Swallowing	Chewing	Oral	Impotency
*Chenopodium murale* L.	Yekusel ketil	Amaranthaceae	H	W and C	L	F	Pounded the leaves and then tied on wounds	Put on	Pounding	Dermal	Wound
*Actiniopteris semiflabellata*	Letef eshok	Pteridaceae	H	W	R	D	The root is crushed and kept in cloth and then tied to the center of the stomach by cotton rope The root is crushed and applied to the affected boy part	Put on Put on	Crushing Crushing	Dermal Dermal	Diarrhea for child A wound caused by venom
*Hyperthelia dissuluta*	Nech sar	Poaceae	H	W	R	F	The root of *Verbascum sinaiticum*, *Aloe pulcherrima,* and *Eleusine floccifolia* is cut into three equal parts and then keep all in the skin of a goat and hang on the neck	Put on neck	Cutting	Dermal	Shotelaye
*Myrica salicifolia* A. Rich.	Shinet	Myricaceae	T	W	R	D	Dried and powdered then smoke it	Fumigating	Powdering	Nasal	Evil eye
*Otostegia fruticosa*	Geram tunjit	Lamiaceae	S	W	L	F	Pound the leaves and mixed them with water and drank	Drinking	Pounding	Oral	Ascariasis
*Dichrostachys cinerea*	Ader	Fabaceae	S	W	L	F	Crushed, squeezed, and rubbed on affected parts	Painting	Pounding	Dermal	Skin allergy caused by spider-like insect
*Pisum sativum*	Ater	Fabaceae	H	C	S	D	Boiled the seed and kept in ventilated cloth and inhaled it through the mouth and nose	Sniffing	Boiling	Nasal and oral	Common cold
*Trichodesma zeylanicum* (Brum.f.) R. Br.	Etse hiwote	Boraginaceae	H	W	R	F	Pounded the fresh root and mixed with water and drank	Drinking	Pounding	Oral	Stomach parasite
*Rumex abyssinicus* Jacq.	Mekemeko	Polygonaceae	H	W	R	F	Pound the root of *Rumex abyssinicus* and leaves of *Inula confertiflora* and then paint the affected body part with seven lemons for seven days	Painting	Pounding	Dermal	Skin disease kuakucha

Habitat (H: herb, T: tree, S: shrub, C: climber); habitat (C: cultivated, W: wild); part used (Bu: bulb, Ba: bark, F: fruit, L: leaf, R: root, S: seed, Wp: whole part); cocondition of preparation (F: fresh, D: dry).

**Table 9:** Medicinal plants which are marketable.

Scientific name	Common name	Use
*Ruta chalepensis*	Tenadam	Spice
*Capsicum annuum*	Karia	Spice and food
*Allium sativum*	Nech shinekurte	Spice and food
*Lippia adoensis*	Koseret	Spice
*Echinops kebericho*	Keberecho	Fragrance
*Rhamnus prinoides*	Gesho	Beverage
*Ziziphus spina-christi*	Geba (qurequra)	Food (fruit) and firewood
*Thymus schimperi*	Tosign	Medicine
*Nigella sativa*	Tekur azemude	Food and spice
*Citrus aurantifolia*	Lomi	Food
*Olea europaea*	Woyera	Construction and to smoke beverage material
*Zingiber officinale*	Zenegebel	Spice
*Vicia faba*	Bakila	Food
*Cicer arietinum*	Shinbera	Food
*Myrtus communis*	Adese	Spice
*Otostegia integrifolia*	Tunget	“Matent”: smoke beverage material
*Trigonella foenumgraecum*	Abish	Food and spice
*Lycopersicon esculentum*	Timatim	Food
*Linum usitatissimum*	Teleba	Food
*Carthamus tinctorius*	Suf	Food
*Sesamum orientale*	Selite	Food
*Lepidium sativum*	Feto	Food
*Brassica carinata*	Gomenezer	Food
*Guizotia abyssinica*	Noog	Food
*Lens culinaris*	Miser	Food
*Coffea arabica*	Bunna	Stimulate
*Brassica nigra*	Senafech	Food
*Cymbopogon citratus*	Tej sar	Fragrance

## Data Availability

The data used to support the findings of this study are included in the article.
